# Quantitative assessment of washout in hepatocellular carcinoma using MRI

**DOI:** 10.1186/s12885-016-2797-9

**Published:** 2016-09-29

**Authors:** Roman Kloeckner, Daniel Pinto dos Santos, Karl-Friedrich Kreitner, Anne Leicher-Düber, Arndt Weinmann, Jens Mittler, Christoph Düber

**Affiliations:** 1 Department of Diagnostic and Interventional Radiology, Johannes Gutenberg-University Medical Centre, Langenbeckst.1, 55131 Mainz, Germany; 2 Department of Internal Medicine, Johannes Gutenberg-University Medical Centre, Langenbeckst.1, 55131 Mainz, Germany; 3 Department of General, Visceral and Transplant Surgery, Johannes Gutenberg-University Medical Centre, Langenbeckst.1, 55131 Mainz, Germany

**Keywords:** Liver Cirrhosis, Liver Neoplasms, Hepatocellular Carcinoma, Magnetic Resonance Imaging, Decision Support Techniques

## Abstract

**Background:**

Arterial hyperenhancement and washout on computed tomography and magnetic resonance imaging (MRI) are described by all major guidelines as specific criteria for non-invasive diagnosis of hepatocellular carcinoma (HCC). However, publications on the quantitative assessment of washout in MRI are lacking. Therefore, we evaluated a method for quantitatively measuring and defining washout in MRI in order to determine a cutoff value that allows objective HCC diagnosis.

**Methods:**

We analyzed all patients who underwent liver transplantation for cirrhosis or liver resection for HCC at our institution between 2003 and 2014. Washout was quantitatively investigated by placing a 25-mm^2^ region of interest (ROI) over each nodule and two 25-mm^2^ ROIs over adjacent liver parenchyma. The percentage signal ratio (PSR = 100 × ratio of signal intensity of adjacent liver to that of the lesion) was calculated for each series in both groups. Accordingly, this quantitative measurement was compared to a qualitative approach.

**Results:**

A total of 16 hypervascularized non-HCC nodules and 69 HCC nodules were identified. Interobserver reliability was reasonably good for the measurement of PSRs and readers showed a substantial agreement for the qualitative assessment. In the HCC group, the median PSR was 116.2 at equilibrium and 112.9 in the delayed phase. In the non-HCC group, the median PSR was 93.8 at equilibrium and 96.0 in the delayed phase. Receiver operating characteristic analysis indicated areas under the curve of 0.902 (*p* < 0.001) and 0.873 (*p* < 0.001) at equilibrium and in the delayed phase. PSR values of 102 at equilibrium and 101.5 in the delayed phase led to the highest Youden’s index of 0.82 and 0.77, respectively. These PSR cutoffs yielded sensitivities of 82 and 77 %, respectively, with specificities of 100 %. The sensitivity for the qualitative assessment of washout was 88 and 93 % and the specificity was 48 and 56 %. For the classification of HCC, sensitivity yielded 95 and 97 % and specificity was 68 and 56 %, respectively.

**Conclusion:**

Quantitatively measuring HCC washout in MRI is easy and reproducible. It can objectify and support diagnosis of HCC. However, the quantitative measurement of washout can only serve as one of several components of HCC assessment.

## Background

Hepatocellular carcinoma (HCC) is one of the most common cancers, with around 750,000 new cases diagnosed annually worldwide [1, 2]. The incidence continues to increase, mainly due to the still increasing numbers of hepatitis B virus and hepatitis C virus infections [3–5]. As most patients with HCC suffer from liver cirrhosis and HCC exhibits specific enhancement on imaging due to the liver’s dual blood supply, the diagnosis of HCC has been simplified considerably. Arterial hyperenhancement and washout of contrast media on portal venous or delayed phase imaging of nodules >1 cm in size enable a final diagnosis of HCC without biopsy in cirrhotic patients based on all major guidelines, such as those from the American Association for the Study of Liver Diseases, the European Association for the Study of the Liver, and the National Comprehensive Cancer Network [1, 6, 7]. The Liver Imaging Reporting and Data System, a classification system for hepatic nodules, also relies primarily on the washout of suspicious nodules [8].

Nonetheless, the evidence for current practice is weak. Washout is highly specific for HCC detection [9–11], but the decision of whether a certain nodule exhibits washout or not is based solely on the subjective impression of the radiologist. Only Liu et al. [12] quantitatively assessed washout and provided a cutoff value for multiphase computed tomography (CT). Although most authors consider magnetic resonance imaging (MRI) superior to CT for HCC imaging [9, 13–15], published studies quantitatively assessing washout in MRI are lacking. Therefore, the purpose of the current investigation was to quantitatively define washout in contrast-enhanced dynamic MRI in order to provide a cutoff value that allows objective HCC diagnosis.

## Methods

### Patients and study design

This study was approved by the responsible ethical body. The need for written consent was waived due to the retrospective analysis of clinical data. Patient records and information were anonymized and de-identified prior to analysis.

We recruited two patient groups: patients with hypervascularized non-HCC nodules and patients with histology-confirmed HCC. Therefore, we selectively searched our database for all patients treated between 2003 and 2014; patients treated before 2003 lacked suitable MRI and mainly underwent MR-angiography without dynamic phase imaging or imaging without fat saturation.

The non-HCC nodule group was recruited from all patients who underwent liver transplantation at our institution. Additional inclusion criteria were proof of cirrhosis in the final pathology report, no proof of HCC in the final pathology report, hypervascular lesion visible on pre-operative MRI, dynamic phase imaging with additional delayed phase imaging (Table 1), and sufficient image quality. The HCC nodule group was not recruited from liver transplant patients. Instead, in order to ensure that the lesion mentioned in the pathology report was identical to the one measured in the MRI, we assembled this group from a population of patients who underwent resection at our institution. To correlate the respective lesions without any doubt, we further restricted our recruitment to patients with postoperative cross-sectional imaging, which allowed us to confirm that the measured lesion was within the resected liver specimen. Additional inclusion criteria were proof of cirrhosis in the final pathology report, proof of HCC in the final pathology report, lesion visible on pre-operative MRI, dynamic phase imaging with additional delayed phase imaging (Table 1), sufficient image quality and nodules in the pathology report that were unequivocally correlated to nodules visualized on pre-operative MRI and postoperative cross-sectional imaging.

**Table 1:** Detailed imaging parameters of the magnetic resonance imaging instruments used in the current investigation

Sonata®	Avanto®	Trio®	Skyra®
T2w^a^ tra	T2w^a^ tra	T2w^a^ tra	T2w^a^ tra
T1w in/opposed phase ^b^ tra	T1w in/opposed phase ^b^ tra	T1w in/opposed phase ^b^ tra	T1w in/opposed phase ^b^ tra
Diffusion-weighted imaging tra	Diffusion-weighted imaging tra	Diffusion-weighted imaging tra	Diffusion-weighted imaging tra
4x T1w fs (native, arterial, portal venous, equilibrium) ^c^	4x T1w fs (native, arterial, portal venous, equilibrium) ^d^	4x T1w fs (native, arterial, portal venous, equilibrium) ^d^	4x T1w fs (native, arterial, portal venous, equilibrium) ^d^
T1w ^b^ tra/cor (delayed)	T1w ^b^ tra (delayed)	T1w ^b^ tra (delayed)	T1w ^b^ tra (delayed)

*tra* transversal, *cor* coronal

^a^ T2-weighted half-Fourier acquisition single-shot turbo spin-echo sequence (HASTE)

^b^ T1-weighted fat-suppressed fast low-angle shot gradient echo sequence (FLASH®)

^c^ T1-weighted fat-suppressed multi-phase contrast-enhanced series (FL 3d)

^d^ T1-weighted fat-suppressed multi-phase contrast-enhanced series (VIBE®)

### MRI and contrast medium

MRI was performed with different scanners: 1.5 T Sonata®, 1.5 T Avanto®, 3 T Trio®, or 3 T Skyra® (all Siemens Healthcare, Germany). All patients underwent a similar imaging protocol comprised of four dynamic, contrast-enhanced, T1-weighted fat saturated, three-dimensional acquisitions and a delayed T1-weighted fat saturated transversal acquisition. More detailed imaging parameters are given in Table 1.

The arterial, portal venous, equilibrium and delayed phases started 20, 45, 90 and 150–180 s, respectively, after the administration of contrast material using a power injector (Accutron MR®; Medtron, Germany). The contrast agents were Magnevist® (gadolinium-diethylenetriaminepentacetate; Bayer Schering Pharma AG, Germany) or Dotarem® (gadolinium-1,4,7,10-tetraazacyclododecane-1,4,7,10-tetraacetic acid; Guerbet, France). Patients investigated with liver-specific contrast agents, such as Primovist® (gadoxetate disodium; Bayer Schering Pharma AG, Germany) were excluded due to entirely different contrast characteristics on delayed-phase imaging.

### Quantitative image analysis

**Fig 1:**
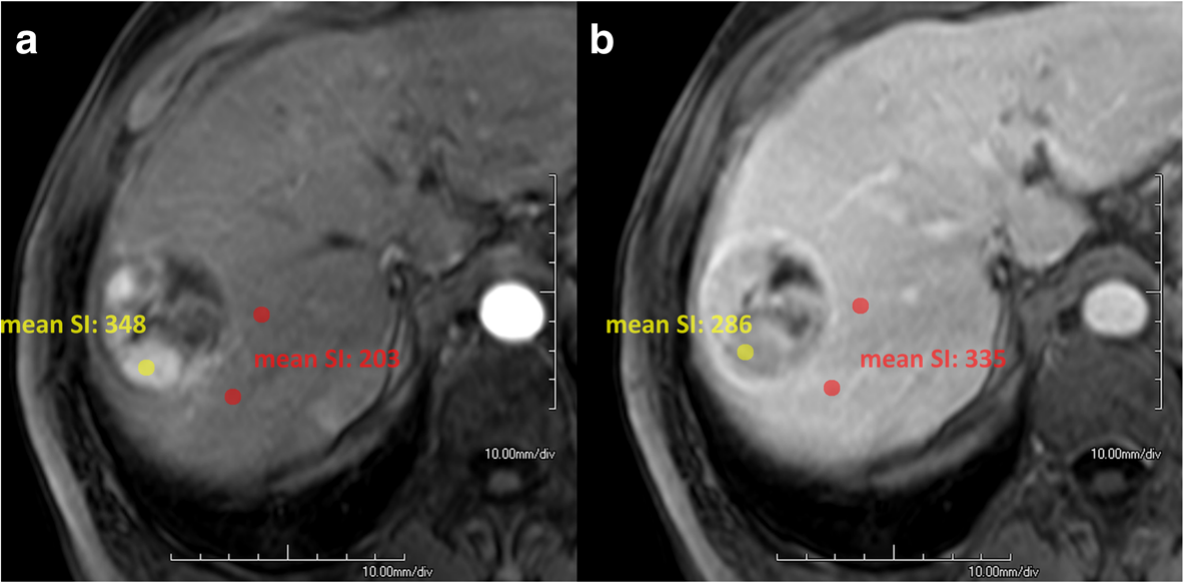
Measurement of signal intensities in a nodule containing hypervascular and non-hypervascular parts due to necrosis. T1- weighted fat-suppressed images in the (**a**) arterial phase and (**b**) equilibrium phase. The lesion ROI (*yellow*) was placed over the hypervascular part. Two identical ROIs were placed over the adjacent liver parenchyma outside the nodule (*red*) in order to average the signal intensity

Hypervascular nodules were identified visually in consensus by two board-certified radiologists with several years of experience in cross-sectional HCC imaging. Nodules already presenting as hyperintense in the native phase were excluded. The diameter of each nodule was recorded and a 25-mm^2^ circular region of interest (ROI) drawn manually over the nodule. The mean signal intensity (SI) was documented by both radiologists in separate evaluation sessions. A maximum of three nodules could be evaluated in a single patient. If a nodule had hypervascular and non-hypervascular parts, e.g., due to necrosis, then the ROI was placed over the hypervascular part. Subsequently, two identical ROIs were placed over the adjacent liver parenchyma outside the nodule (Fig. 1) and the SIs of these ROIs averaged.

Liu et al. [12] calculated the percentage attenuation ratio, attenuation change and relative washout ratio, concluding that the percentage attenuation ratio was the most useful parameter for differentiating between HCC and non-HCC. In our case, calculating a value analogous to the percentage attenuation ratio seemed reasonable because the SI in MRI is not an absolute measurement, but an arbitrary value. As MRI measures the SI instead of X-ray attenuation, we named this proportional measure the percentage signal ratio (PSR). The PSR was calculated for all contrast-enhanced phases using the following formula:

$$ \mathrm{P}\mathrm{S}\mathrm{R}=100\times \left(\mathrm{A}\mathrm{S}/\mathrm{L}\mathrm{S}\right) $$

where AS (adjacent SI) corresponds to the average of two areas adjacent to the lesion and LS is the SI of the lesion. ROIs were placed at corresponding coordinates in all phases.

### Qualitative image analysis

The visually identified nodules were evaluated independently in a separate session by two blinded readers experienced in cross-sectional HCC imaging. The readers were asked to classify nodules as having washout or not and if suspicious or not suspicious for HCC. The results of this qualitative evaluation were compared to the quantitative approach described above.

### Pathology

All explanted livers were sectioned into parallel 5-10-mm slices. Afterwards, specimens from suspicious areas were stained for further investigation by microscopy. In unclear cases, additional staining and procedures were performed at the pathologist’s discretion to obtain a final diagnosis.

### Computational and statistical analysis

For ROI placement and SI measurement, we used Aquarius.NET Viewer® version 4.4.8.85 (Terarecon, USA). Primary data collection and PSR calculations were carried out in Excel® 2013 (Microsoft Corporation, USA). Statistical analyses were performed in SPSS® version 22 (IBM Corporation, USA). Interobserver variability for categorical variables was measured by calculating kappa values and using the intraclass correlation coefficient for interval variables. Groups were compared using the Mann-Whitney *U* test as the distribution of PSR exhibited skewness in some settings. The significance level was chosen as α = 0.05. Receiver operating characteristic (ROC) analysis was performed using the dedicated ROC-curve tool in SPSS. To determine the optimal cutoff value we calculated Youden’s index for each given PSR value in each imaging phase [16].

### Results

**Fig 2:**
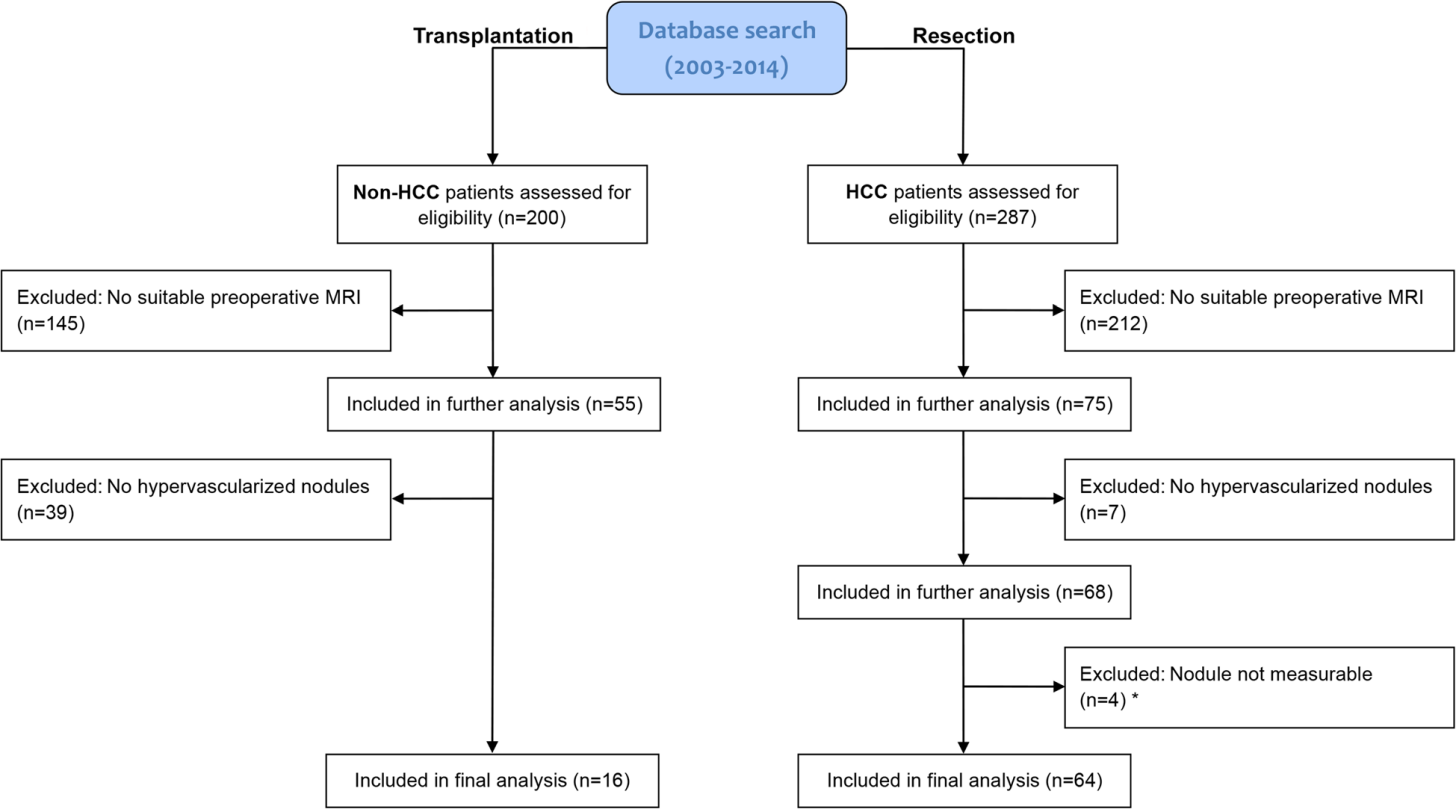
Flowchart of patient inclusion in the two study groups. *Four patients were excluded from the resection/HCC group due to non-measurable lesions because of diffuse tumor (*n* = 2 patients) and the presence of additional adenoma in the resected specimen (*n* = 2 patients). Therefore, an unequivocal lesion-to-lesion correlation between MRI and the pathology report was not possible

Initially, the liver transplantation group included 200 patients and the resection group 287 patients. A total of 184 and 223 patients were excluded from the respective groups for various reasons. Thus, the final number of patients in the liver transplantation and resection groups were 16 and 64, respectively (Fig. 2). In the liver transplantation group, 16 hypervascular non-HCC nodules were analyzed (mean size 15 ± 3.5 mm, range 10–22 mm). In the resection group five patients had two nodules, and a total of 69 HCC nodules were analyzed (mean size 62 ± 45.8 mm, range 11–174 mm). Most tumors were moderately differentiated (G1, *n* = 12; G2, *n* = 49; G3, *n* = 8). The quantitative analysis showed reasonably good interobserver reliability for the computed PSRs in the respective dynamic phases. Intraclass correlation coefficients yielded 0.68, 0.69. 0.72, 0.69 and 0.61 for the native, arterial, portal venous, equilibrium and late dynamic imaging phase, respectively [17].

**Fig 3:**
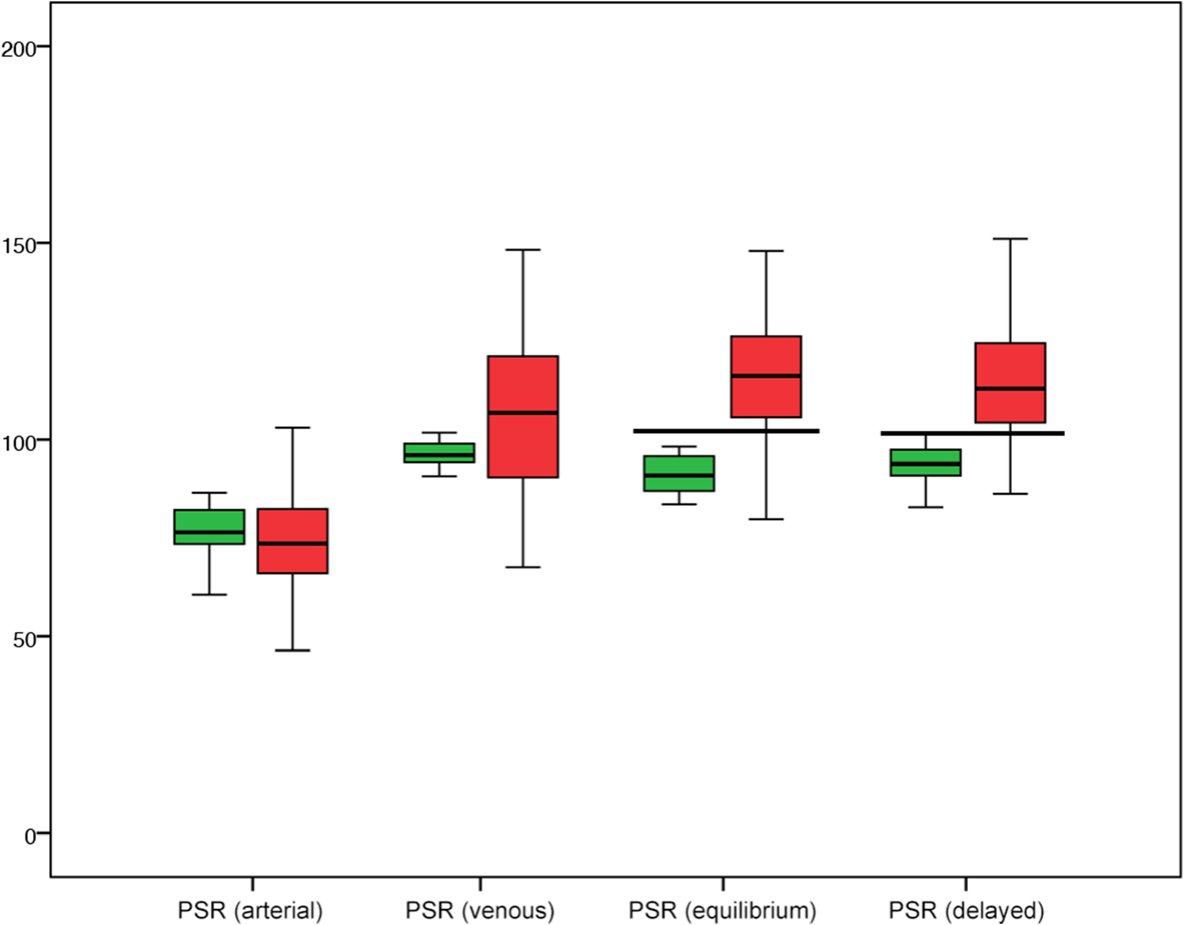
PSRs in HCC and non-HCC nodules in the different contrast phases. The median PSR for non-HCC (*green*) remained <100 in all contrast phases, whereas most lesions in the HCC-group (*red*) exhibited considerable washout, corresponding to a PSR >100. The overlap was smallest in the equilibrium and delayed phases. The horizontal lines indicate cutoff values of 102 and 101.5, which provide the best discrimination between HCC and non-HCC

All visually depicted lesions in the non-HCC group were truly hypervascular according to the PSR. The later the phase, the greater the convergence of the SI of the nodule and the SI of the adjacent liver, yielding median PSRs that approached 100 (76.4, 90.9, 93.8 and 96.0 in the arterial, portal venous, equilibrium and delayed phases, respectively; Table 2 and Fig. 3). Even in the delayed phase, the median PSR was considerably less than 100. Yet, 2 of the 16 non-HCC nodules (12.5 %) had PSRs >100 in the equilibrium phase (101.5, 100.3), meaning that these nodules had slightly lower SIs than the adjacent liver in terms of washout.

**Table 2:** Signal intensities (SIs) over the nodule and adjacent liver parenchyma and the resulting percentage signal ratio (PSR) for each contrast-enhanced phase. Data are presented as median (Q1/Q3)

	Arterial			Portal venous			Equilibrium			Delayed		
	Nodule (SI)	Liver (SI)	PSR	Nodule (SI)	Liver (SI)	PSR	Nodule (SI)	Liver (SI)	PSR	Nodule (SI)	Liver (SI)	PSR
Non-HCC	255.5 (197.8/345.2)	183.7 (158.6/260.9)	76.4 (73.7/81.9)	302.7 (234.8/346.8)	275.6 (207.9/321.3)	90.9 (87.0/95.7)	306.5 (223.3/352.1)	284.1 (201.0/328.6)	93.8 (91.2/97.2)	329.5 (261.3/569.5)	235.2 (195.2/424.8)	96.0 (94.4/98.8)
HCC	211.0 (168.0/291.6)	156.8 (129.5/208.8)	73.6 (66.4/82.2)	249.0 (208.2/303.2)	261.3 (212.9/322.8)	106.8 (90.5/121.0)	229.2 (191.5/283.3)	272.8 (220.1/322.3)	116.2 (105.6/126.1)	251.5 (202.1/309.5)	212.0 (171.4/266.3)	112.9 (104.3/124.4)
p	<0.001			0.233			0.005			<0.001

In the second group, all HCC nodules exhibited substantial hypervascularization (Table 2). A majority of these nodules had SIs in later imaging phases that were markedly lower than the SIs of adjacent liver parenchyma in terms of washout, yielding median PSRs >100 (73.6, 106.8, 116.2 and 112.9 in the arterial, portal venous, equilibrium and delayed phases, respectively; Table 2 and Fig. 3). No washout was observed for 11 of the 69 HCC nodules (15.9 %) in the equilibrium phase and 14 of the 69 HCC nodules (20.2 %) in the delayed phase.

Significantly different PSRs between the HCC and non-HCC groups were found in all but the arterial contrast phase (PSR_native_ 128.3 vs. 94.6, *p* < 0.001; PSR_arterial_ 73.6 vs. 76.4, *p* = 0.344; PSR_venous_ 106.8 vs. 90.9, *p* = 0.004; PSR_equilibrium_ 116.2 vs. 93.8, *p* < 0.001; PSR_delayed_ 112.9 vs. 96.0, *p* < 0.001).

**Fig 4:**
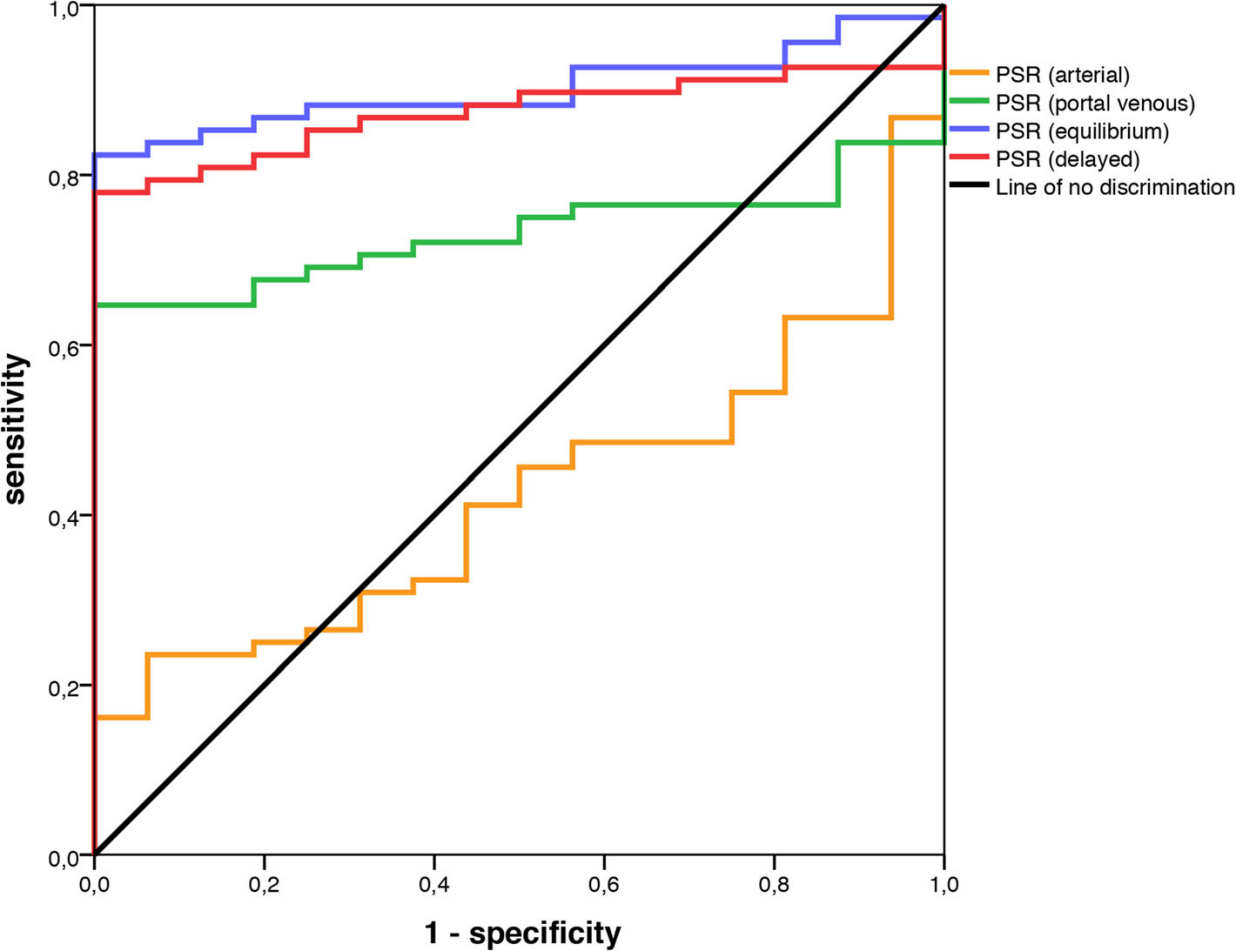
Receiver operating characteristic analysis. The arterial phase provided no discrimination between HCC and non-HCC, with an area under the curve of 0.424 (*p* = 0.344). Portal venous, equilibrium and delayed phases differentiated more effectively, with areas under the curve of 0.732 (*p* = 0.004), 0.902 (*p* < 0.001), and 0.873 (*p* < 0.001), respectively

The ROC analysis clearly indicated that data from the arterial phase were near the “line of no discrimination”, with an area under the curve of only 0.424. The portal venous, equilibrium and delayed phases more effectively discriminated HCC nodules from non-HCC nodules, with areas under the curve of 0.732, 0.902 and 0.873, respectively (Fig. 4). Cutoff values of 102.0 at equilibrium and 101.5 in the delayed phase led to the highest Youden’s index of 0.82 and 0.77, respectively. PSR cutoffs of 102.0 at equilibrium and 101.5 in the delayed phase yielded sensitivities of 82 and 77 %, respectively, with specificities 100 % (Fig. 3).

In the qualitative analysis, both readers showed a substantial level of agreement for the subjective assessment of washout (Kappa 0.698, *p* < 0.001) and the classification of HCC (Kappa 0.672, *p* < 0.001) [18]. Reader 1 correctly classified 64 of 84 nodules as either having washout or no washout, the second reader correctly classified 59 of 84 nodules. This yields a sensitivity of 88 and 93 % and a specificity of 48 and 56 % for washout. In the second step reader 1 classified 76 of 84 nodules correctly as either HCC or non-HCC, and the second reader had a rate of 75 of 84, resulting in a sensitivity and specificity of 95/68 % and 97/56 %, respectively. This corresponds to a Youden’s index of 0.63 and 0.53 for the classification of HCC. Of the two non-HCC nodules exhibiting quantitative washout in equilibrium phase, both were classified as HCC by both readers.

## Discussion

An accurate description of tumor size and nodule number is important for patient management. According to the Milan and United Network of Organ Sharing criteria, only patients with limited tumor load are amenable to liver transplantation. Therefore, surgeons often demand exact discrimination between HCC and non-HCC lesions, especially in patients with severe liver cirrhosis and multiple nodules with different contrast dynamics.

Liu et al. [12] were the first investigators to define a quantitative cutoff for washout in CT. Their suggested cutoff of 107 was chosen to maximize lesion detection and yielded a sensitivity of 100 % and specificity of 75.8 % [12]. In our analysis we sought to maximize the diagnostic performance of the quantitative method and compared it to the qualitative evaluation by two experienced readers. We found that the PSR was significantly different between the HCC and non-HCC group. Therefore, the PSR cutoff values of 102 at equilibrium and 101.5 in the delayed phase could serve as an aid for radiologists to differentiate between HCC and non-HCC, providing even more concise statements for liver surgeons and other clinicians. Due to the resulting specificity and positive predictive values of 100 %, these cutoffs would also be in line with the clinical guidelines for non-invasive diagnosis of HCC [1, 6, 7]. In the future, they could probably be used as one of several components for automatic classification.

This study has several limitations. First, there may be some bias due to small sample size and patient selection, as the non-HCC group was transplanted due to cirrhosis and only had hypervascular lesions that were not prospectively felt to represent HCC. In contrast, the HCC group underwent resection for lesions thought to be HCC. Second, washout is only one of several criteria in the decision of whether a lesion is suspicious for HCC. All lesions in the HCC group that did not exhibit washout were correctly classified as HCC due to other malignancy criteria such as size, pseudocapsula or irregular pattern. However, this is in concordance with the literature, in which around 20 % of HCCs exhibit no washout [19–21]. Third, all explanted livers were investigated by the pathologists according to a standardized procedure and no HCC was found, but in the final gross pathology report not all non-HCC lesions were described in detail, including the two with washout, which were also classified as HCC by both readers in this setting. This leaves some uncertainty as to whether these lesions were dysplastic nodules, vascular malformations or any other type of hepatic lesion. Our primary purpose was to evaluate the methodology for quantification of washout in MRI and all measurements were performed on MRI machines from a single manufacturer using roughly the same imaging protocol. However, the use of different MRI scanners from other manufacturers is unlikely to lead to different results, as PSR is a relative and non-dimensional measure. Furthermore, the exact location of the ROI may have a considerable effect on the measured SI. Nonetheless, by following the instructions for measurement described in the Methods section, the intraclass correlation coefficients in our analysis varied between 0.61 and 0.72 for the respective imaging phases, indicating a reasonably good interobserver reliability. Our late-phase delay varied between 150 and 180 s, which is in concordance with Liu et al. [12] and within the timing suggested by the new policy from the Organ Procurement and Transplantation Network/United Network for Organ Sharing [12, 22].

## Conclusions

This study showed that quantitatively defining washout in MRI of the liver by measuring the PSR is easy and reproducible. We obtained results similar to those of Liu et al. [12] using MRI instead of CT. The PSR cutoff values were 102 and 101.5 at equilibrium and in the delayed phase. This approach can improve and objectify HCC diagnosis. However, the quantitative measurement of washout can only serve as one of several components of HCC assessment. Additional research might be useful to further optimize and validate our approach and set definitive cutoff values derived from a larger sample.

## Abbreviations

HCC: Hepatocellular carcinoma
MRI: Magnetic resonance imaging
ROI: Region of interest
SI: Signal intensity
PSR: Percentage signal ratio
